# Timing of intervention in high-risk non-ST-elevation acute coronary syndromes in PCI versus non-PCI centres: Sub-group analysis of the ELISA-3 trial

**DOI:** 10.1007/s12471-015-0801-7

**Published:** 2016-01-28

**Authors:** E. A. Badings, W. S. Remkes, J-H. E. Dambrink, S. H. K. The, J. Van Wijngaarden, G. Tjeerdsma, S. Rasoul, J. R. Timmer, M. L. J. van der Wielen, D. J. A. Lok, A. W. J. van ’t Hof

**Affiliations:** Deventer Hospital, Deventer, The Netherlands; Isala Klinieken, Dokter van Heesweg 2, 8025AB Zwolle, The Netherlands; Treant Zorggroep location Bethesda, Hoogeveen, The Netherlands; Hospital De Tjongerschans, Heerenveen, The Netherlands; Atrium Medisch Centrum, Heerlen, The Netherlands; Maastricht UMC, Maastricht, The Netherlands

**Keywords:** Non ST-segment elevation acute coronary syndrome, Revascularisation, Timing, Elderly, Clinical outcome, Interventional clinics, Non-interventional clinics

## Abstract

**Aims:**

To compare the effect of timing of intervention in patients with non-ST-elevation acute coronary syndrome (NSTE-ACS) in percutaneous coronary intervention (PCI) versus non-PCI centres.

**Methods and results:**

A post-hoc sub-analysis was performed of the ELISA III trial, a randomised multicentre trial investigating outcome of early (< 12 h) versus late (> 48 h) angiography and revascularisation in 542 patients with high-risk NSTE-ACS. 90 patients were randomised in non-PCI centres and tended to benefit more from an early invasive strategy than patients included in the PCI centre (relative risk 0.23 vs. 0.85 [*p* for interaction = 0.089] for incidence of the combined primary endpoint of death, reinfarction and recurrent ischaemia after 30 days of follow-up). This was largely driven by reduction in recurrent ischaemia. In non-PCI centres, patients randomised to the late group had a 4 and 7 day longer period until PCI or coronary artery bypass grafting, respectively. This difference was less pronounced in the PCI centre.

**Conclusions:**

This post-hoc analysis from the ELISA-3 trial suggests that NSTE-ACS patients initially hospitalised in non-PCI centres show the largest benefit from early angiography and revascularisation, associated with a shorter waiting time to revascularisation. Improved patient logistics and transfer between non-PCI and PCI centres might therefore result in better clinical outcome.

## Introduction

A routinely invasive strategy (angiography and revascularisation if applicable) is recommended by the current guidelines [1, 2] in high-risk patients with non-ST-elevation acute coronary syndrome (NSTE-ACS). The majority of trials and meta-analysis on this topic showed a reduction in the incidence of cardiovascular death and myocardial infarction (MI) in the medium to long term [3–9], although for example the Dutch Ictus study (Invasive versus Conservative Treatment in Unstable Coronary Syndromes [10]) did not. Studies investigating optimal timing of intervention showed conflicting results. Meta-analysis of randomised controlled trials (RCTs) [11, 12] and observational studies [13] comparing early to later timing showed that early timing of intervention does not lead to a reduction in the incidence of death or myocardial infarction but at most reduces the incidence of recurrent ischaemia.

A limitation of these studies is that they were largely conducted in tertiary care, high-volume, percutaneous coronary intervention (PCI) centres. In the Netherlands, as in many other European countries, however, a considerable number of these patients are initially hospitalised in non-PCI centres. After medical stabilisation and coronary angiography, the patient is then transferred to the PCI centre for revascularisation, if needed. This makes generalisability of the study results to clinical practice questionable.

Observational studies [14, 15] showed that patients admitted to a centre with angiography and PCI facilities were more likely to receive an invasive strategy, had a lower risk for refractory or recurrent angina at the cost of higher risk for stroke and major bleeding. No difference was found in the incidence of cardiovascular death, myocardial infarction or stroke at 6 months. According to the investigators, these findings support the strategy of directing patients with suspected ACS to the nearest hospital with acute care, irrespective of the availability of interventional facilities. Because these studies were non-randomised and performed about 15 years ago, when background medication was different from today, these data should be interpreted with caution.

The ELISA 3 trial compared an early versus delayed invasive strategy in a population of high-risk patients hospitalised for NSTE-ACS in 6 centres (5 non-PCI and 1 PCI) in the Netherlands. The aim of the current sub-study is to investigate the clinical effect of timing of invasive strategy in patients randomised in the participating non-PCI centres and to compare this to patients randomised in the PCI centre.

## Methods

The ELISA-3 study was an investigator-initiated, randomised, open, multicentre study. Rationale, design and results have been published previously [16]. Patients were eligible if they were hospitalised with ischaemic chest pain or dyspnoea at rest and had at least 2 out of 3 of the following high-risk characteristics: (1) evidence of extensive myocardial ischaemia on ECG (shown by new cumulative ST depression > 5 mm or temporary ST-segment elevation in 2 contiguous leads < 30 min), (2) elevated biomarkers (troponin T > 0.10 μg/l or myoglobin > 150 μg/l) or elevated creatine kinase-myocardial band (CK-MB) fraction (> 6 % of total CK) and (3) age above 65 years. Randomisation had to take place within 24 h of the last episode of ischaemic symptoms. Exclusion criteria were persistent ST-segment elevation, symptoms of ongoing myocardial ischaemia despite optimal medical therapy, contraindication for diagnostic angiography, active bleeding, cardiogenic shock, acute posterior infarction and life expectancy less than 1 year. The trial was conducted in six Dutch hospitals of which one had 24-hour facilities for (primary) PCI and coronary artery bypass grafting (CABG). The study was registered in the ISRCTN Register (ISRCTN39230163).

### Patients

After giving written informed consent, patients were randomly assigned to an immediate or delayed treatment strategy. In patients assigned to the immediate treatment strategy, angiography was performed as soon as possible but within 12 h of randomisation. Patients assigned to the delayed treatment strategy underwent angiography no sooner than 48 h after randomisation unless, despite optimal medical therapy, clinical instability or recurrent ischaemia warranted emergency angiography. Patients recruited at a non-PCI centre and randomised to the immediate intervention group were urgently transferred to the PCI centre for angiography and subsequent revascularisation; in case of assignment to delayed intervention, angiography was performed at the non-PCI centre and the patient was transferred to the PCI centre for intervention if necessary. All patients were treated according to the current guidelines.

### Endpoints

Primary endpoint of the ELISA-3 study as well for the current sub-analysis was the combined incidence of all-cause mortality, re-infarction and/or recurrent ischaemia at 30-day follow-up. Secondary endpoints were enzymatic infarct size as assessed by a single cardiac troponin T, at 72–96 h after admission or at discharge and the percentage of patients without a rise in CK-MB during admission. In addition, bleeding complications were assessed. Major bleeding was defined as bleeding with a haemoglobin drop of ≥ 2 mmol/l or a blood transfusion of 2 or more units. All endpoints were adjudicated by an independent endpoint committee.

### Statistical analysis

Data were analysed according to the intention-to-treat analysis. Continuous variables were expressed as median and interquartile range (IQR) and were compared between the intervention groups using a Mann-Whitney U test. Categorical data were described by proportions and compared with the Chi square or Fisher’s exact test. Logistic regression was used to calculate the *p*-value of the interaction between the effect of the intervention and the subgroups upon the primary endpoint. All tests were two-sided and an alpha of 5 % was used. Statistical analysis was performed with SPSS (version 20).

## Results

### Comparison of PCI vs. non-PCI centres

Of the total number of 542 patients in the trial, 444 (82 %) were included in the PCI centre and 90 in one of the 5 non-PCI centres. Eight patients were excluded, 5 for withdrawn consent and 3 for major protocol violations. Baseline characteristics and clinical outcome are shown in Table 1. Patients in non-PCI centres were older and there were less smokers. Time from admission to randomisation was comparable. Time from randomisation to start of angiography was longer in the non-PCI centres (23.8 vs. 15.1 h, *p* = 0.07) as was time from angiography to PCI (0.75 vs. 0.37 h, *p* < 0.001) and angiography to CABG (122.8 vs. 189.9 h, *p* = 0.037). No difference was found in the incidence of the primary endpoint between the two groups or in the individual components. The percentage of patients without a rise in CK-MB during admission was significantly higher in patients from the non-PCI centres (52.6 vs. 33.1 %, *p* = 0.01). Incidence of bleeding events was comparable.

**Table 1:** Baseline characteristics, study endpoints and clinical outcomes of patients randomised in PCI versus non-PCI centres

		PCI centre (*n* = 444)	Non-PCI centres (*n* = 90)	*P*-value
Demographics	Age (years, IQR)	71.19 (62.9–78.26)	73.51 (67.84–78.41)	0.017
	Male gender, *n* (%)	302/444 (68.0 %)	59/90 (65.6 %)	0.649
GRACE score	(median, IQR)	136 (117–154)	135 (119–151)	0.877
Medical history (*n*,%)	Hypertension	248/444 (55.9 %)	52/90 (57.8 %)	0.738
	Smoking	113/444 (25.5 %)	14/90 (15.6 %)	0.044
	Diabetes	102/444 (23.0 %)	16/90 (17.8 %)	0.279
	Previous MI	84/444 (18.9 %)	16/90 (17.8 %)	0.800
	Previous TIA	22/444 (5.0 %)	6/90 (6.7 %)	0.447
	Previous stroke	20/444 (4.5 %)	1/90 (1.1 %)	0.228
	Previous PCI	89/444 (20.0 %)	15/90 (16.7 %)	0.461
	Previous CABG	59/444 (13.3 %)	10/90 (11.1 %)	0.574
Time (hours, median, IQR)	Admission—randomisation	2.05 (1.11–4.11)	2.16 (0.08–6.36)	0.910
	Randomisation angiography	15.13 (2.29–54.07)	23.83 (2.83–70.05)	0.070
	Angiography—PCI	0.37 (0.18–0.65)	0.75 (0.30-93.25)	< 0.001
	Angiography—CABG	122.78 (49.93–230.85)	189.92 (96.78–325.27)	0.037
	Angiography—revascularisation	0.47 (0.23–43.66)	69.17 (0.43-190.45)	< 0.001
Extent CAD	One vessel	113/434 (26.0 %)	27/86 (31.4 %)	0.494
	Two vessel	144/434 (23.2 %)	24/86 (27.9 %)	
	Three vessel	127/434 (29.3 %)	28/86 (32.6 %)	
Infarct-related artery	LAD	133/382 (34.8 %)	34/79 (43.0 %)	0.340
	RCA	81/382 (21.2 %)	15/79 (19.0 %)	
	Circumflex	90/382 (23.6 %)	22/79 (23.6 %)	
	Left main	7/382 (1.8 %)	1/79 (1.3 %)	
	Graft	34/382 (8.9 %)	4/79 (5.1 %)	
Treatment	PCI	254/384 (66.1 %)	44/79 (55.7 %)	0.140
	CABG	87/384 (22.7 %)	26/79 (32.9 %)	
	Medical	43/384 (11.2 %)	9/79 (11.4 %)	
Primary endpoint (%)	Combined endpoint^a^	52/436 (11.9 %)	11/87 (12.6 %)	0.851
	Death	5/436 (1.1 %)	1/87 (1.1 %)	> 0.99
	MI	5/436 (1.1 %)	2/87 (2.3 %)	0.329
	Recurrent ischaemia	44/436 (10.1 %)	9/87 (10.3 %)	0.943
Secondary endpoints	Enzymatic infarct size^b^	0.31 (0.11–0.86)	0.30 (0.11–0.73)	0.918
	% patients without CKMB rise	147/444 (33.1 %)	40/76 (52.6 %)	0.001
Bleeding	Any bleeding	93/436 (21.3 %)	19/87 (21.8 %)	0.916
	Major bleeding	52/436 (11.9 %)	8/87 (9.2 %)	0.465
	CABG-related bleeding	79/436 (18.1 %)	14/87 (16.1 %)	0.652
	CABG-related major bleeding	47/436 (10.8 %)	7/87 (8.0 %)	0.444

*CABG* coronary artery bypass graft, *CAD* coronary artery disease, *CKMB* creatine kinase-MB, *IQR* inter quartile range, *LAD* left anterior descending, *MI* myocardial infarction, *PCI* percutaneous coronary intervention, *RCA* right coronary artery, *TIA* transient ischaemic attack.

^a^Combined primary endpoint = incidence of death, reinfarction and recurrent ischaemia at 30-day follow-up.

^b^Single troponin T 72–96 h after admission (µg/l, median, IQR).

### Effect of early vs. delayed treatment in patients included in non-PCI centres

Of the 90 patients who were included in a non-PCI centre, 45 (50 %) were randomised to immediate treatment and 45 to a delayed invasive treatment strategy. Baseline characteristics were largely comparable (Table 2), with exception of gender and percentage of smokers. As intended by the protocol, median time from randomisation to angiography was 3.0 h in the immediate and 70 h in the delayed treatment group. In addition, time from the start of the coronary angiography to revascularisation (PCI or CABG, if any) was 0.7 vs. 146 h; for PCI 0.37 vs. 96.5 h [4.0 days] and for CABG 115 [4.8 days] vs. 275 h [11.5 days]. The combined primary endpoint occurred in 4.7 % of the patients in the immediate treatment group and 20.5 % of the patients in the delayed treatment group (*p* = 0.027). This difference was driven by a reduction in the occurrence of recurrent ischaemia in the immediately treated patients (2.3 vs. 18.2 %, *p* = 0.03 in the immediate and delayed treatment group respectively).The incidence of the secondary endpoints and bleeding events did not differ.

**Table 2:** Baseline characteristics, study endpoints and clinical outcome of patients randomised in non-PCI centres

		Early treatment (*n* = 45)	Late treatment (*n* = 45)	*P*-value
Demographics	Age (years, IQR)	73.78 (69.97–78.41)	72.97 (67.55–77.97)	0.377
	Male gender, *n* (%)	35/45 (77.8 %)	24/45 (53.3 %)	0.015
GRACE score	(median, IQR)	136 (129–157)	130 (113–149)	0.406
Medical history (*n*,%)	Hypertension	24/45 (53.3 %)	28/45 (62.2 %)	0.393
	Smoking	3/45 (6.7 %)	11/45 (24.4 %)	0.020
	Diabetes	6/45 (13.3 %)	10/45 (22.2 %)	0.270
	Previous MI	5/45 (11.1 %)	11/45 (24.4 %)	0.098
	Previous TIA	3/45 (6.7 %)	3/45 (6.7 %)	> 0.99
	Previous stroke	0/45 (0.0 %)	1/45 (2.2 %)	> 0.99
	Previous PCI	6/45 (13.3 %)	9/45 (20.0 %)	0.396
	Previous CABG	6/39 (13.3 %)	4/45 (8.9 %)	0.502
Time (hours, median, IQR)	Admission – randomisation	2.8 (0.08–5.84)	1.87 (0.06–9.06)	0.994
	Randomisation – angiography	3.04 (2.23–4.03)	70.05 (50.6–112.58)	< 0.001
	Angiography – PCI	0.37 (0.24–0.73)	96.52 (29-146.28)	< 0.001
	Angiography – CABG	114.75 (42.05–219.53)	275.25 (175.57–494.15)	0.012
	Angiography-revascularisation	0.71 (0.29–63.69)	146.28 (82.04–296.96)	< 0.001
Extent CAD	One vessel	15/44 (34.1 %)	12/42 (28.6 %)	0.531
	Two vessel	10/44 (22.7 %)	14/42 (33.3 %)	
	Three vessel	14/44 (31.8 %)	14/42 (33.3 %)	
Infarct-related artery	LAD	18/39 (46.2 %)	16/40 (40.0 %)	0.461
	RCA	7/39 (17.9 %)	8/40 (20.0 %)	
	Circumflex	9/39 (23.1 %)	13/40 (32.5 %)	
	Left main	0/39	1/40 (2.5 %)	
	Graft	2/39 (5.1 %)	2/40 (5.0 %)	
Treatment	PCI	24/39 (61.5 %)	20/40 (50.0 %)	0.685
	CABG	11/39 (28.2 %)	15/40 (37.5 %)	
	Medical	4/39 (10.3 %)	5/40 (12.5 %)	
Primary endpoint (%)	Combined endpoint^a^	2/43 (4.7 %)	9/44 (20.5 %)	0.027
	Death	0/43 (0.0 %)	1/44 (2.3 %)	> 0.99
	MI	1/43 (2.3 %)	1/44 (2.3 %)	> 0.99
	Recurrent ischaemia	1/43 (2.3 %)	8/44 (18.2 %)	0.030
Secondary endpoints (%)	Enzymatic infarct size^b^	0.36 (0.13–0.73)	0.29 (0.05–1.10)	0.538
	% patients without CKMB rise	22/44 (50.0 %)	18/32 (56.3 %)	0.590
Bleeding	Any bleeding	9/43 (20.9 %)	10/44 (22.7 %)	0.839
	Major bleeding	4/43 (9.3 %)	4/44 (9.1 %)	> 0.99
	CABG-related bleeding	6/43 (14.0 %)	8/44 (18.2 %)	0.592
	CABG-related major bleeding	3/43 (7.0 %)	4/44 (9.1 %)	> 0.99

*CABG* coronary artery bypass graft, *CAD* coronary artery disease, *CKMB* creatine kinase-MB, *IQR* inter quartile range, *LAD* left anterior descending, *MI* myocardial infarction, *PCI* percutaneous coronary intervention, *RCA* right coronary artery, *TIA* transient ischaemic attack.

^a^Combined primary endpoint = incidence of death, reinfarction and recurrent ischaemia at 30 day follow-up.

^b^Single troponin T 72–96 h after admission (µg/l, median, IQR).

### Effect of timing of intervention in patients from PCI centre vs. non-PCI centres

**Fig. 1:**
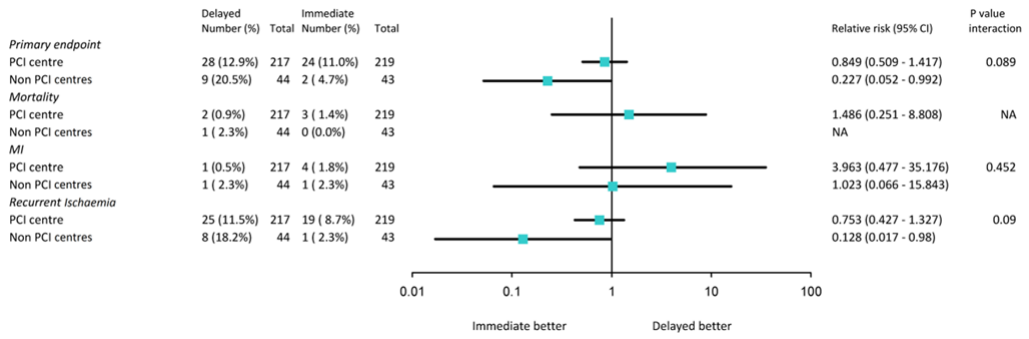
Forest plot of relative risk of primary and secondary endpoints at 30 days of follow-up in patients randomised to early or late intervention in a PCI centre versus non-PCI centres. Data are numbers or percentages, unless otherwise indicated. Percentages are number of events divided by number of patients. Squares and horizontal bars represent within-subgroup relative-risk and 95 % CIs, respectively, on a log scale

The effect of the timing of the intervention on the primary endpoint is shown in Fig. 1. Patients included in a non-PCI centre tended to benefit more from an early invasive strategy than those included in the PCI centre (relative risk 0.23 vs. 0.85) but this difference was not statistically significant (*p*-value for interaction = 0.089) and largely driven by reduction in recurrent ischaemia. No differences were found in the incidence of secondary endpoints and bleeding.

## Discussion

This sub-study, comparing the effect of timing of intervention in high-risk non-ST-elevation acute coronary syndromes between patients hospitalised in PCI and non-PCI centres shows that non-PCI centre patients tend to benefit more from an early invasive strategy, mainly due to reduction in recurrent ischaemia. These results were found in relationship to a much larger difference in revascularisation times between early and late treated patients in the patients admitted primarily in a non-PCI centre.

**Fig. 2:**
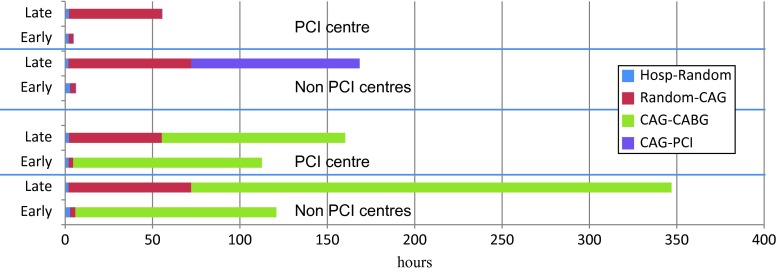
Timing of procedures in PCI and non-PCI-centres for patients randomised to early or late intervention. *Hosp* hospitalisation, *random* randomisation, *CAG* coronary angiography, *PCI* percutaneous coronary intervention

In patients initially hospitalised in non-PCI centres, median time from coronary angiography to revascularisation was longer in those randomised to a delayed strategy compared with an early invasive strategy (Fig. 2). This was caused by a difference in patient logistics: patients randomised to an immediate strategy were urgently transferred to the PCI centre where the coronary angiography and—when appropriate—in the same procedure a PCI was performed. Patients randomised to a delayed invasive strategy underwent a coronary angiography in the non-PCI centre and underwent a PCI later on, after their angiogram had been discussed with the heart team consisting of interventional cardiologists and thoracic surgeons, and transfer to the PCI centre. This resulted in an additional waiting time after angiography of 4 days for PCI and even 7 days for CABG. For patients randomised in the PCI centre, this difference did not occur, because all patients underwent coronary angiography and PCI (if feasible) in the same procedure, irrespective of the group they were randomised to. Median waiting time for CABG in patients hospitalised in the PCI centre was 123 h (5.1 days), comparable with patients randomised to the early invasive strategy in non-PCI centres.

**Fig. 3:**
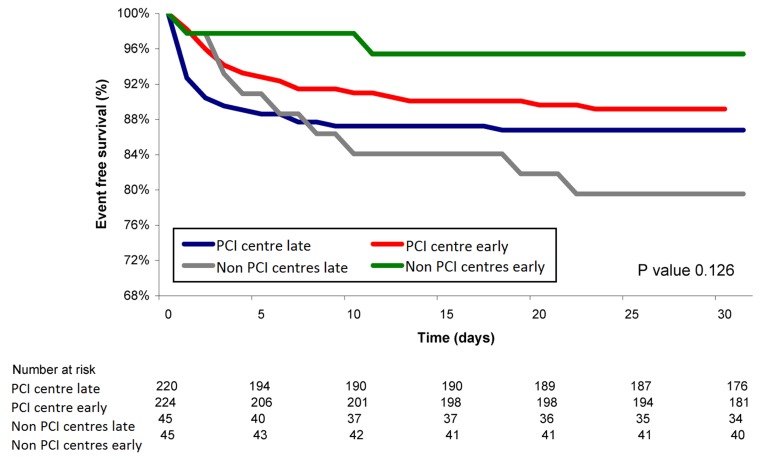
Kaplan-Meyer curves for event-free survival from recurrent ischaemia for each of the 4 treatment groups

This difference in logistics of patients with NSTE-ACS between PCI and non-PCI centres also exists in daily practice. In PCI centres, angiography and revascularisation are generally performed in the same procedure, while patients in non-PCI centres first undergo a coronary angiography, then are discussed with the team of the PCI centre and are scheduled for revascularisation a couple of days later. In the meantime, these patients are prone to developing recurrent ischaemic symptoms, as can been seen in the Kaplan-Meyer curve of this group of patients for event-free survival from recurrent ischaemia in the first week (Fig. 3). Considering this, shortening the time between angiography and revascularisation might reduce the recurrence of ischaemia and improve clinical outcome in patients initially hospitalised in a non-PCI centre. Agreements with PCI centres to assure short access time for revascularisation and application of information technologies for fast distant evaluation of angiograms may be helpful. Periodic national audits, as for example snapshot registries of the National Cardiovascular Data Registry (NCDR), could be used to monitor the achievement of pre-determined quality indicators. Because the role of the ECG in initial ACS triage is limited [17], other factors should also be taken into account with the assessment of urgency for revascularisation.

As far as we know, the ELISA 3 trial is the first randomised trial investigating the effect of timing of intervention in high-risk NSTE-ACS patients that included patients in both PCI and non-PCI centres, giving the opportunity to compare outcome in these two types of centres. No significant differences in clinical outcome were found, showing that initial medical stabilisation and angiography in non-PCI centres is safe and feasible.

This study has the following limitations. Clinical characteristics of both patient groups differ. For example, slightly more patients in the PCI centre had no significant coronary artery disease (11.5 vs. 8.1 %) which might have influenced our findings. Further, this sub-study is based on a post-hoc sub-group analysis with a relatively small number of patients. Therefore, our findings should be interpreted with caution. From our data, the cause of the trend towards a higher risk of recurrent ischaemia in patients randomised to delayed invasive strategy in non-PCI clinics cannot be proven. Therefore, our hypothesis that this might be caused by a longer time interval between angiography and revascularisation needs further investigation. If new studies were to confirm our hypothesis, a change in the management of this large group of patients might be considered.

The main finding of the ELISA-3 study was that in high-risk NSTE-ACS patients, early angiography and revascularisation led to a non-significant 30 % relative risk reduction (RRR) for the combined endpoint of death, re-infarction or recurrent ischaemia at 30 days, driven by a reduction in recurrent ischaemia (RRR = 40 %, *p* = 0.058). These results are largely consistent with previously conducted randomised trials and meta analyses [9–11] and show that immediate intervention is not associated with a reduction in hard clinical endpoints (death or myocardial infarction) but at most reduces the incidence of recurrent ischaemia. This post-hoc analysis shows that the sub-group of patients initially hospitalised in non-PCI centres especially benefit from an early invasive strategy. A plausible explanation is the shorter waiting time between angiography and revascularisation in these patients.
